# Patient safety culture in Austria and recommendations of evidence-based instruments for improving patient safety

**DOI:** 10.1371/journal.pone.0274805

**Published:** 2022-10-17

**Authors:** Šehad Draganović, Guido Offermanns

**Affiliations:** 1 Department of Organization, Human Resources, and Service Management, Faculty of Management and Economics, University of Klagenfurt, Klagenfurt am Wörthersee, Austria; 2 Karl Landsteiner Society, Institute for Hospital Organization, Vienna, Austria; Aga Khan University pakistan, PAKISTAN

## Abstract

This study aimed to investigate the patient safety culture in Austria. We identified factors that contributed to a higher degree of patient safety and subsequently developed evidence-based suggestions on how to improve patient safety culture in hospitals. Moreover, we examined differences in the perception of patient safety culture among different professional groups. This study used a cross-sectional design in ten Austrian hospitals (N = 1,525). We analyzed the correlation between ten patient safety culture factors, three background characteristics (descriptive variables), and three outcome variables (patient safety grade, number of adverse events reported, and influence on patient safety). We also conducted an analysis of variance to determine the differences in patient safety culture factors among the various professional groups in hospitals. The findings revealed that all ten factors have considerable potential for improvement. The most highly rated patient safety culture factors were communication openness and supervisor/manager’s expectations and actions promoting safety; whereas, the lowest rated factor was non-punitive response to error. A comparison of the various professional groups showed significant differences in the perception of patient safety culture between nurses, doctors, and other groups. Patient safety culture in Austria seems to have considerable potential for improvement, and patient safety culture factors significantly contribute to patient safety. We determined evidence-based practices as recommendations for improving each of the patient safety factors.

## Introduction

The report "To Err is Human: Building a Safer Health System" estimated that annually in the United States of America, deaths caused by medical errors ranged from 44,000 to 98,000 patients [1]. After the publication of this report, similar studies were conducted in Europe (France [2], England [3], Sweden [4], the Netherlands [5–7], Spain [8], Portugal [9], and the Republic of Irland [10]) using the same research methodology–the Harvard Medical Practice Study. The rate of adverse events (AEs) in these European countries ranged from 5.7% to 12.3%. Between 20% and 70% of these events were preventable adverse events (PAEs). Moreover, PAEs that contributed to death ranged from 0.25% to 7.4%. Studies to assess patient safety were also conducted using other research methods, such as the Global Trigger Tool. These studies showed that approximately 13.5%– 33.2% of hospitalized patients experienced an AE and from the total number of AEs, between 44% and 63.1% were PAEs. The estimated mortality was between 0.4% and 1% [11–13].

Many risk management tools (e.g., surgical safety checklist, handoff program, etc.) have been developed to reduce the incidence of AEs. Relevant studies have demonstrated that appropriate risk management tools significantly reduce AEs [14–19]. However, in some settings and countries, these measures have not led to the desired improvements [20, 21]. This problem arose because the tools were not embedded in the hospital’s culture or were neglected shortly after their introduction. Attempts have been made to solve this problem unilaterally by changing the structures and processes of the hospital. However, improving patient safety cannot focus solely on changing structures (e.g., introducing an error reporting system) and processes (e.g., introducing surgical checklists); rather, a change in the safety culture must be implemented and achieved [22–24]. Safety culture has been defined as “the product of individual and group values, attitudes, perceptions, competencies, and patterns of behavior that determine the commitment to, and the style and proficiency of, an organization’s health and safety management [25]”.

Safety culture is the key to successfully implementing risk management tools or improving patient safety [26, 27]. Assessing the status of the existing safety culture in a hospital has been identified as the first step in the development of a strong safety culture [26, 28]. The current state of safety culture has not yet been measured in Austria–a gap that needs to be addressed. Therefore, the primary goal of this study was to measure safety culture to ascertain the current situation. Specifically, this study examined how patient safety factors affect overall patient safety. In addition, comparisons were made between the different professional groups. The secondary goal of this study was to define evidence-based recommendations for improvements in Austrian hospitals based on the data obtained.

## Materials and methods

### The Austrian Patient Safety Climate Inventory

The study was conducted using the Austrian Patient Safety Climate Inventory” (A-PaSKI), a newly developed and psychometrically verified instrument [29]. This was developed based on Austrian empirical data and showed very good results at the model, indicator, and construct levels. The A-PaSKI has a total of 30 items clustered into ten different factors. Seven unit-level factors (supervisor/manager’s expectations and actions promoting safety, teamwork within units, communication openness, feedback and communication about error, non-punitive response to error, unit management support for patient safety, and unit handoffs and transitions), two hospital-level factors (hospital management support for patient safety and hospital handoffs and teamwork across hospital units), and one outcome factor (frequency of event reporting). All items were scored on a 5-point Likert scale (“strongly disagree” to “strongly agree”) or frequency (“never” to “always”). The A-PaSKI includes several demographic background variables, eight of which were used in the analysis, along with three outcome variables (patient safety grade, number of events reported, and influence on patient safety) [29].

### Design and setting

This study was conducted in ten Austrian hospitals, with all professional groups, departments, wards, and areas involved in the survey. The data collection in each hospital lasted four weeks, and the overall data collection lasted a total of seven months from August 2016 to February 2017. The questionnaire was distributed to all staff present at the time, resulting in 1,525 participants. We also defined 18 work areas and six staff roles within these work areas.

All data were analyzed with SPSS Statistics 24.0 (Superior Performing Software System) and AMOS 24.0 (Analysis of Moment Structures). The study was approved by the Committee for Scientific Integrity and Ethics (Ethics Committee) of the Danube Private University (DPU) (GZ: DPU-EK/019). Participants gave their verbal consent to participate. Written consent was not obtained because only anonymous data was collected. The study did not contain personal data and could not be linked to them. All relevant information about the study was explained, including its nature, purpose, risks, potential benefits, available alternatives, and the opportunity to ask questions and opt out if necessary. Participants were informed that they had the freedom, without coercion or undue influence, not to participate in this study and to withdraw at any time. The invitation to the survey contained a statement confirming the anonymity and confidentiality of the participants. Consent information was documented by emailing to all participants before the survey. In addition, the online questionnaire included consent information. The ethics committees of the Danube Private University approved this consent process.

### Data analysis

First, all background variables (response rate, hospital or clinic size, staff position in the hospital, period in current hospital, period worked in the current unit, percentage of employment [part-time/full-time/specific percentage], contact with patients, and period in the current profession) were elaborated descriptively. Subsequently, the ordinally scaled variables (5-point Likert scale) were considered as metrical ones, and the following was first illustrated: Valid frequencies (N), Mean (M), Standard deviation (SD), Confidence interval (CI), Skewness (Sk), and Kurtosis (Ku). All mean values below three were interpreted as negative, mean values of three as neutral, and mean values greater than three were interpreted as positive values. Therefore, the factors and variables between unit, hospital, and outcome levels were distinguished. Then, the three individual outcome variables were elaborated descriptively. Within the framework of inferential statistics, the Spearman correlation was calculated with ten factors, three outcome and three background variables, and tested for significance.

In the analysis of variance (ANOVA), all professional groups were compared based on the factors’ mean values. Thus, in the first step, the ANOVA test and Welch’s test were used to check whether the ten hospitals differed in the ten factors. In order to determine whether there were significant differences between the professional groups, the Tukey HSD post hoc test and the Tamhane post hoc test were used in the second step to compare the mean values [30]. In the first three columns, the absolute frequency (N), mean values (M), and confidence intervals (CIs) were calculated for each group. In the penultimate column, the one-way ANOVA, the Tukey HSD post hoc test was marked with the letter "a" and the Welch test Tamhane post hoc test was marked with two small letters "ab." In the last column, the group means were tested with the post hoc test; they differed significantly at an alpha level of 0.05 and are marked by capital letters.

## Results

### Overall patient safety in Austria

A total of 1,525 employees participated in the survey, amounting to a response rate of 25.1%. The lowest and highest response rates were 18.2% and 32.1%, respectively (see Table 1). We conducted a nonresponse bias technique (archival analysis) to check for potential sample bias [31]. We compared respondents (professional groups) to non-respondents (professional groups) on variables contained in an archival database from Statistik Austria [32]. We found no significant differences in the composition of our sample and hospital staff in Austria.

**Table 1:** Response rate and hospital size.

Hospital	Response rate	Hospital size
Hospital A	30.7%	Small
Hospital B	26.3%	Small
Hospital C	19.3%	Large
Hospital D	30.3%	Medium
Hospital E	19.7%	Small
Hospital F	18.2%	Small
Hospital G	32.1%	Medium
Hospital H	27.8%	Small
Hospital I	24.0%	Small
Hospital J	22.5%	Large
Total	25.1%	/

The response rate of individual hospitals showed that anesthesiology and intensive care with 10%, internal medicine with 9.2%, and orthopedics with 9.5% were best represented (see Table 2). Nearly 10% of the doctors, 46.9% of the nurses and other health workers, 13% of the medical technicians and therapists, 6.6% of the administrative professionals, and 2% from other professional groups participated in the survey. From the total number of respondents, 21.6% did not specify which professional group they belonged to (see Table 2 for this and other background information on the survey).

**Table 2:** Clinics and background information for A-PaSKI.

Variable	Category	N	N%
**What is your primary work area or unit in this hospital? **	Anesthesiology and intensive care	152	10.0%
	Accident surgery	97	6.4%
	Roentgen	26	1.7%
	Laboratory	37	2.4%
	Surgery	108	7.1%
	Internal Medicine	141	9.2%
	Neurology	44	2.9%
	Orthopedics	145	9.5%
	Psychiatry/mental health	71	4.7%
	Pneumology	27	1.8%
	Radio-oncology	25	1.6%
	Ophthalmology	29	1.9%
	Dermatology	35	2.3%
	Gynecology	54	3.5%
	Ear-nose-throat diseases	26	1.7%
	Pediatric Medicine	85	5.6%
	Administration	57	3.7%
	Other	325	21.3%
	No response	41	2.7%
	Total	1525	100.0%
**What is your staff position in this hospital?**	Doctor/specialist/assistant	151	9.9%
	Nurse/Registered Nurse	661	43.3%
	Other health workers	55	3.6%
	Medical technicians and therapists	198	13.0%
	Management/administrative professions	100	6.6%
	Other	30	2.0%
	No response	330	21.6%
	Total	1525	100.0%
**How long have you worked in this hospital?**	Less than three months	18	1.2%
	Less than one year	48	3.1%
	One to five years	253	16.6%
	More than five years	912	59.8%
	No response	294	19.3%
	Total	1525	100.0%
**How long have you worked in your current hospital work area/unit?**	Less than three months	20	1.3%
	Less than one year	70	4.6%
	One to five years	319	20.9%
	More than five years	811	53.2%
	No response	305	20.0%
	Total	1525	100.0%
**How many percent do you work in this hospital?**	Full-time, 100%	797	67.2%
	85%	33	2.8%
	75%	151	12.7%
	62.5%	48	4.0%
	50%	126	10.6%
	Part-time, 25%	31	2.6%
	Total	1186	100.0%
**In your staff position, do you typically have direct interaction or contact with patients?**	Yes	1095	71.8%
	No	116	7.6%
	No response	314	20.6%
	Total	1525	100.0%
**How long have you worked in your current specialty or profession? **	Less than one year	39	2.6%
	One to five years	216	14.2%
	More than five years	965	63.3%
	No response	305	20.0%
	Total	1525	100.0%

N–Absolute frequencies, N%–Relative frequencies.

After a detailed description of the sample, the results of the general perception of the safety culture were presented on three levels (seven unit-level factors, two hospital-level factors, and one outcome factor) (see Table 3). The mean values of the six unit-level factors ranged from 3.58 to 3.76, and only for one unit-level factor, non-punitive response to error, the mean value was almost at the limit of 3.0. The CI results indicated that in the population, the mean values of the six unit-level factors were in the positive range. However, the results of the individual variables can differ. For example, for the unit-level factor teamwork within units, the variable people support one another in this unit is in the negative range. The same factor delivers significantly better results for the variable when a lot of work needs to be done quickly, we work together as a team to get the work done.

**Table 3:** Descriptive statistics for A-PaSKI (Factors and individual items).

					95% CI			
Unit-level factors and variable	Cronbach’s α	N	M	SD	-	+	Sk	Ku
**Supervisor/manager’s expectations and actions promoting safety**	**0.795**	**1169**	**3.73**	**0.80**	**3.69**	**3.78**	**-0.711**	**0.540**
My supervisor/manager says a good word when he/she sees a job done according to established patient safety procedures		1315	3.62	1.06	3.45	3.79	-0.708	0.045
My supervisor/manager seriously considers staff suggestions for improving patient safety		1281	3.93	0.99	3.76	4.09	-0.889	0.590
Whenever pressure builds up, my supervisor/manager wants us to work faster, even if it means taking shortcuts		1288	3.76	0.92	3.61	3.90	-0.836	0.864
My supervisor/manager overlooks patient safety problems that happen over and over		1224	3.98	0.87	3.84	4.12	-1.344	2.626
**Teamwork within units**	**0.773**	**1488**	**3.58**	**0.611**	**3.54**	**3.61**	**-0.694**	**0.064**
People support one another in this unit		1514	2.80	0.40	2.73	2.86	-1.484	0.204
When a lot of work needs to be done quickly, we work together as a team to get the work done		1506	4.30	0.74	4.18	4.42	-0.649	-0.560
In this unit, people treat each other with respect		1505	3.90	0.75	3.78	4.02	-0.032	-0.745
**Communication openness**	**0.672**	**1170**	**3.76**	**0.774**	**3.72**	**3.81**	**-0.633**	**0.197**
Staff feel free to question the decisions or actions of those with more authority		1229	3.65	0.95	3.50	3.81	-0.425	-0.490
Staff are afraid to ask questions when something does not seem right		1234	3.94	1.02	3.77	4.10	-0.951	0.516
In this unit, we discuss ways to prevent errors from happening again		1270	4.07	0.96	3.91	4.22	-0.791	-0.139
**Feedback and communication about error**	**0.759**	**1184**	**3.58**	**0.990**	**3.52**	**3.63**	**-0.488**	**-0.382**
We are given feedback about changes put into place based on event reports		1211	3.74	1.07	3.57	3.92	-0.612	-0.496
We are informed about errors that happen in this unit		1259	3.97	0.87	3.82	4.11	-0.627	0.134
**Non-punitive response to error**	**0.741**	**1224**	**3.18**	**0.623**	**3.15**	**3.22**	**0.246**	**0.808**
Staff feel like their mistakes are held against them		1428	3.31	0.92	3.16	3.46	-0.237	0.056
When an event is reported, it feels like the person is being written up, not the problem		1382	3.61	0.91	3.46	3.76	-0.698	0.529
Staff worry that mistakes they make are kept in their personnel file		1192	3.68	1.01	3.52	3.85	-0.683	0.107
**Unit management support for patient safety**	**0.841**	**966**	**3.60**	**0.892**	**3.54**	**3.65**	**-0.597**	**0.221**
Unit management provides a work climate that promotes patient safety		1289	3.78	0.98	3.62	3.94	-0.471	-0.191
Unit management has a clear picture of the risk associated with patient care		1166	3.83	1.04	3.66	4.00	-0.813	0.262
Unit management considers patient safety when program changes are discussed		1092	3.85	0.91	3.70	4.00	-0.751	0.686
The actions of unit management show that patient safety is a top priority		1375	3.90	1.01	3.74	4.07	-0.699	-0.044
**Unit handoffs and transitions**	**0.769**	**1021**	**3.59**	**0.753**	**3.55**	**3.64**	**-0.572**	**0.949**
Important patient care information is often lost during shift changes		1214	3.66	0.88	3.52	3.80	-0.621	0.544
Shift changes are problematic for patients in this unit		1157	3.99	0.72	3.87	4.10	-0.646	0.786
Things “fall between the cracks” when transferring patients in the unit		1129	3.54	0.87	3.40	3.69	-0.549	0.055
**Hospital-level factors and variable**								
**Hospital management support for patient safety**	**0.871**	**1034**	**3.32**	**1.045**	**3.25**	**3.38**	**-0.397**	**-0.580**
Hospital management provides a work climate that promotes patient safety		1195	3.81	0.99	3.65	3.97	-0.643	0.171
The actions of hospital management show that patient safety is a top priority		1112	3.92	0.95	3.76	4.07	-0.766	0.448
Hospital management seems interested in patient safety only after an adverse event happens		1092	3.63	1.09	3.45	3.81	-0.761	-0.131
**Hospital handoffs and teamwork across hospital units**	**0.719**	**1062**	**3.42**	**0.742**	**3.37**	**3.47**	**-0.532**	**0.736**
Problems often occur in the exchange of information in unit		1337	3.24	0.93	3.09	3.39	-0.494	-0.084
It is often unpleasant to work with staff from other hospital units		1130	3.72	0.79	3.59	3.85	-0.296	-0.237
**Outcome factor and variable**								
**Frequency of event reporting**	**0.883**	**892**	**3.31**	**1.078**	**3.24**	**3.38**	**-0.235**	**-0.841**
When a mistake is made, but is caught and corrected before affecting the patient, how often is this reported?		966	3.61	1.11	3.42	3.79	-0.608	-0.334
When a mistake is made, but has no potential to harm the patient, how often is this reported?		953	3.16	1.10	2.98	3.34	-0.109	-0.897
When a mistake is made that could harm the patient, but does not, how often is this reported?		937	3.52	1.16	3.33	3.71	-0.512	-0.658

N–Valid frequencies, M–Mean, SD–Standard deviation, CI–Confidence interval, Sk–Skewness, Ku–Kurtosis. Data in bold are total data for each factor.

The results of the two factors at the hospital level were within the positive range. It can be seen that all variables are in the positive range. The mean results of the outcome factors and their individual variables could be easily explained and checked with other outcome variables. Thus, the mean value of the frequency of event reporting was 3.31, which is just above the limit of three. It is clear that AEs are very rarely reported when no patients have been harmed (variable: when a mistake is made, but has no potential to harm the patient, how often is this reported?). To check the results of this factor, we considered the outcome variables (number of events reported in the past twelve months, patient safety grade and influence on patient safety) for A-PaSKI (see Table 4). It is particularly notable that 68% of the respondents did not report a single AE in the last 12 months. Among the respondents, 22.8% reported one or two events and only 9.2% of the respondents reported three or more events (see first row in Table 4).

**Table 4:** Outcome variables for A-PaSKI.

Variable	Category	N	N%
**Number of events reported in the past twelve months**	No event reports	790	68.0%
	One to two event reports	265	22.8%
	Three to five event reports	65	5.6%
	Six to ten event reports	25	2.2%
	Eleven to 20 event reports	12	1.0%
	21 event reports or more	4	0.4%
	Total	1161	100.0%
**Patient safety grade**	Excellent	101	7.2%
	Very good	743	53.0%
	Acceptable	432	30.8%
	Poor	58	4.1%
	Failing	25	1.8%
	No response	43	3.1%
	Total	1402	100.0%
**Influence on patient safety**	Strongly disagree	20	1.4%
	Disagree	71	5.1%
	Neither	284	20.4%
	Agree	568	40.8%
	Strongly agree	377	27.1%
	No response	73	5.2%
** **	Total	1393	100.0%

N–Absolute frequencies, N%–Relative frequencies.

A total of 7.2% of health professionals felt that the level of patient safety in their hospitals was excellent, 53% were of the opinion that safety was very good, 30.8% saw it as acceptable, and just under 6% were of the opinion that patient safety was poor. The possibility of influencing patient safety is a prerequisite for improving patient safety. The results showed that 67.9% of the staff can influence patient safety, 20.4% of them can do so only partially, whereas 6.5% of the staff cannot influence patient safety at all (see Table 4).

### Correlations between patient safety factors and outcome variables

Spearman correlation coefficients were calculated to determine the strength of the relationship between factors and outcome variables and between factors and duration of employment or working hours (see Table 5). There was a highly positive correlation between the outcome variable patient safety grade and the factors supervisor/manager’s expectations and actions promoting safety (rs = 0.53), unit management support for patient safety (rs = 0.65) as well as hospital management support for patient safety (rs = 0.64). This means that the stronger the support from supervisors, department heads, and hospital managers, the better the level of patient safety. A mean correlation was observed between patient safety grade and teamwork within units (rs = 0.36), communication openness (rs = 0.41), feedback and communication about error (rs = 0.44), non-punitive response to error (rs = 0.30), and frequency of event reporting (rs = 0.36).

**Table 5:** Inferential statistics with original 10 factors.

	Patient safety grade	Influence on patient safety	Number of events reported	Period in current hospital	Period worked in current unit	Percent of employment
**Unit-level**						
Supervisor/ manager expectations and actions promoting safety	0.530*** (N = 1159)	0.244*** (N = 1115)	-0.134*** (N = 1006)	-0.012 (N = 1059)	-0.03 (N = 1052)	0.126*** (N = 1021)
Teamwork within units	0.361*** (N = 1331)	0.205*** (N = 1297)	-0.035 (N = 1137)	-0.083** (N = 1205)	-0.066* (N = 1195)	0.019 (N = 1162)
Communication openness	0.411*** (N = 1152)	0.242*** (N = 1114)	-0.004 (N = 1035)	0.001 (N = 1084)	-0.009 (N = 1078)	0.07* (N = 1046)
Feedback and communication about error	0.444*** (N = 1165)	0.229*** (N = 1120)	-0.049 (N = 1048)	0.059 (N = 1101)	0.037 (N = 1095)	0.04 (N = 1063)
Non-punitive response to error	0.307*** (N = 1184)	0.132*** (N = 1161)	-0.066* (N = 1014)	-0.094** (N = 1068)	-0.069* (N = 1061)	0.052 (N = 1032)
Unit management support for patient safety	0.651*** (N = 953)	0.361*** (N = 938)	-0.103**(N = 814)	0.007 (N = 853)	0.007 (N = 849)	0.043 (N = 827)
Unit handoffs and transitions	0.469*** (N = 1004)	0.162*** (N = 981)	-0.109** (N = 862)	-0.009 (N = 904)	-0.003 (N = 898)	0.048 (N = 878)
***Hospital-level***						
Hospital management support for patient safety	0.649*** (N = 1025)	0.277*** (N = 990)	-0.08* (N = 960)	-0.035 (N = 1004)	-0.007 (N = 1000)	0.008 (N = 976)
Hospital handoffs and Teamwork across hospital units	0.345*** (N = 1051)	0.19*** (N = 1014)	-0.101** (N = 982)	0.045 (N = 1030)	0.053 (N = 1024)	0.067* (N = 999)
***Outcome***						
Frequency of event reporting	0.367*** (N = 884)	0.161*** (N = 859)	0.086* (N = 816)	0.08* (N = 840)	0.102** (N = 835)	0.019 (N = 821)

* p = 0.05

** p = 0.01

*** p = 0.001

The second outcome variable, influence on patient safety, correlated only slightly with the new patient safety culture (PSC) factors. A low correlation can be found with unit management support for patient safety (rs = 0.36). The third outcome variable, number of events reported, and all three demographic variables (period in current hospital, period worked in current unit, and percent of employment) did not show any significant correlations with the PSC factors.

### Comparison of different professional groups

Based on the mean values, the most profound differences between professional groups was seen between physicians and nursing staff (see Table 6). The statistical significance of these differences was calculated using an ANOVA with five different professional groups. The last column shows the professional groups that differ significantly. For supervisors/managers’ expectations and actions promoting safety, it is obvious that nurses, medical technicians, and therapists as well as management rated their supervisors’ performance in promoting patient safety significantly better than doctors. In other words, doctors are more critical of supervisors than other professional groups.

**Table 6:** A-PaSKI factors–Comparison between professional groups.

					95% CI			
Factor	Group	N	M	SD	-	+	ANOVA/ Welch	Post-Hoc^C^
**Supervisor/ manager’s expectations and actions promoting safety**	Doctor/specialist/assistant (A)	132	3.50	0.072	3.36	3.64	F(4.1004) = 10.563. p = .002 ^a^	BDE
	Nurse/Registered Nurse (B)	608	3.75	0.032	3.69	3.82		
	Other health workers (C)	46	3.60	0.114	3.37	3.83		
	Medical technicians and therapists (D)	156	3.79	0.064	3.67	3.92		
	Management/ administrative professions (E)	67	3.89	0.089	3.71	4.07		
** **	**Total**	**1009**	**3.73**	**0.025**	**3.68**	**3.78**		
**Teamwork within units**	Doctor/specialist/assistant (A)	149	3.43	0.052	3.33	3.53	F(4.1140) = 6.522. p = .001^a^	BD
	Nurse/Registered Nurse (B)	650	3.63	0.023	3.59	3.68		
	Other health workers (C)	55	3.53	0.079	3.37	3.69		
	Medical technicians and therapists (D)	194	3.65	0.043	3.57	3.74		
	Management/ administrative professions (E)	97	3.52	0.066	3.38	3.65		
** **	**Total**	**1145**	**3.60**	**0.018**	**3.56**	**3.63**		
**Communication openness**	Doctor/specialist/assistant (A)	134	3.62	0.071	3.48	3.76	F(4.1035) = 5.227. p = .066 ^a^	
	Nurse/Registered Nurse (B)	619	3.81	0.031	3.75	3.88		
	Other health workers (C)	46	3.87	0.108	3.65	4.09		
	Medical technicians and therapists (D)	168	3.73	0.058	3.62	3.84		
	Management/ administrative professions (E)	73	3.70	0.086	3.53	3.88		
** **	**Total**	**1040**	**3.77**	**0.024**	**3.72**	**3.82**		
**Feedback and communication about error**	Doctor/specialist/assistant (A)	137	3.57	0.083	3.41	3.73	F(4.1050) = 3.080. p = .530 ^a^	
	Nurse/Registered Nurse (B)	626	3.56	0.040	3.49	3.64		
	Other health workers (C)	46	3.70	0.131	3.43	3.96		
	Medical technicians and therapists (D)	166	3.53	0.073	3.38	3.67		
	Management/administrative professions (E)	80	3.73	0.114	3.50	3.96		
** **	**Total**	**1055**	**3.58**	**0.030**	**3.52**	**3.64**		
**Non-punitive response to error**	Doctor/specialist/assistant (A)	132	3.16	0.059	3.05	3.28	F(4.1009) = 2.224. p = .220^a^	
	Nurse/Registered Nurse (B)	593	3.17	0.026	3.12	3.22		
	Other health workers (C)	49	3.14	0.058	3.03	3.26		
	Medical technicians and therapists (D)	157	3.29	0.052	3.18	3.39		
	Management/ administrative professions (E)	83	3.12	0.061	3.00	3.25		
** **	**Total**	**1014**	**3.18**	**0.020**	**3.14**	**3.22**		
**Unit management support for patient safety**	Doctor/specialist/assistant (A)	125	3.61	0.081	3.45	3.77	F(4.820) = 14.635. p = .001 ^a^	
	Nurse/Registered Nurse (B)	501	3.50	0.040	3.42	3.58		E
	Other health workers (C)	36	3.78	0.114	3.55	4.01		
	Medical technicians and therapists (D)	104	3.73	0.088	3.55	3.90		
	Management/ administrative professions (E)	59	3.94	0.106	3.72	4.15		
** **	**Total**	**825**	**3.59**	**0.031**	**3.53**	**3.65**		
**Unit handoffs and transitions**	Doctor/specialist/assistant (A)	128	3.33	0.077	3.18	3.48	F(4.874) = 12.421. p = .001 ^a^	BCDE
	Nurse/Registered Nurse (B)	590	3.62	0.029	3.57	3.68		
	Other health workers (C)	41	3.72	0.113	3.49	3.94		
	Medical technicians and therapists (D)	80	3.64	0.084	3.47	3.80		
	Management/administrative professions (E)	40	3.83	0.134	3.55	4.10		
** **	**Total**	**879**	**3.59**	**0.025**	**3.54**	**3.64**		
**Hospital management support for patient safety**	Doctor/specialist/assistant (A)	130	3.29	0.085	3.12	3.45	F(4.963) = 42.644. p = .001 ^a^	E
	Nurse/Registered Nurse (B)	588	3.16	0.044	3.07	3.24		DE
	Other health workers (C)	42	3.60	0.158	3.28	3.91		
	Medical technicians and therapists (D)	142	3.55	0.075	3.41	3.70		
	Management/ administrative professions (E)	66	3.82	0.127	3.57	4.08		
** **	**Total**	**968**	**3.30**	**0.033**	**3.23**	**3.36**		
**Hospital handoffs and Teamwork across hospital units **	Doctor/specialist/assistant (A)	140	3.30	0.072	3.16	3.44	F(4.993) = 5.134. p = .050^a^	D
	Nurse/Registered Nurse (B)	601	3.41	0.029	3.35	3.47		
	Other health workers (C)	39	3.53	0.105	3.31	3.74		
	Medical technicians and therapists (D)	151	3.55	0.056	3.44	3.66		
	Management/ administrative professions (E)	67	3.39	0.096	3.20	3.58		
** **	**Total**	**998**	**3.42**	**0.023**	**3.37**	**3.46**		
**Frequency of event reporting **	Doctor/specialist/assistant (A)	113	3.17	0.092	2.98	3.35	F(4.804) = 21.110. p = .001 ^a^	C
	Nurse/Registered Nurse (B)	502	3.23	0.049	3.14	3.33		C
	Other health workers (C)	32	3.84	0.167	3.50	4.18		
	Medical technicians and therapists (D)	115	3.43	0.095	3.24	3.61		
	Management/ administrative professions (E)	47	3.65	0.159	3.33	3.97		
	**Total**	**809**	**3.30**	**0.038**	**3.23**	**3.37**		

N–Valid frequencies, M–Mean, SD–Standard deviation, CI–Confidence interval

^a^ Equal variances assumed: One-Way ANOVA, Tukey HSD Post-Hoc test

^ab^ Equal variances not assumed: Welch’s test, Tamhane Post-Hoc test

^C^. letter represents an independent group with significantly higher mean value

^D^. Data in bold are total data for each factor.

A similarly critical attitude emerges in terms of teamwork within units. Here, nurses, medical technicians, and therapists rated teamwork significantly better than doctors. Regarding the factors of communication openness, feedback, communication about error, and non-punitive response to error, no significant differences were found. Unit management support for patient safety was rated significantly better by management and administrative professions than by nurses. All professional groups (BCDE) perceived unit handoffs and transitions as significantly better than doctors did.

However, the post hoc test results revealed that nurses and doctors rated hospital management support for patient safety significantly better than management/administrative professionals. Nurses rated this factor significantly better than medical technicians and therapists. Doctors also indicated a significantly more critical perception of hospital handoffs and teamwork across hospital units than other health professionals. The frequency of event reporting yielded particularly interesting results, and all other professional groups reported more events in the error reporting system than doctors and nurses did.

## Discussion

This study evaluated the PSC in ten Austrian hospitals. Overall, all PSC factors showed considerable potential for improvement on the unit, hospital, and outcome levels. At the unit level, six factors (supervisor/manager’s expectations and actions promoting safety, teamwork within units, communication openness, feedback and communication about error, unit management support for patient safety, and unit handoffs, and transitions) showed an acceptable value. These results are comparable to those of other studies [33–35]. The non-punitive response to error factor showed an unacceptable value, which is also comparable to another study [36].

Supervisors/manager’s expectations and actions promoting safety, and unit management support for patient safety were very important factors; without management support, it is almost impossible to establish risk management tools such as the WHO checklist [16, 17]. Therefore, the Institute for Healthcare Improvement has defined leadership as a key success factor for patient safety in its paper [27]. The low scores for these factors may be explained by insufficient education on safety and inappropriate leading style.

The results regarding the factor teamwork within units can perhaps be explained by non-existent team training in hospitals. That is, team practice outside real-world situations is negligible. Studies have shown the relationship between teamwork within units and clinical patient outcomes, including error rates and patient mortality [37, 38]; therefore, Austrian hospitals should implement team training to improve teamwork within units. A meta-analysis showed that team training may improve healthcare [39]. For instance, Austrian hospitals could use crew resource management, medical team training, and team strategies and tools to enhance performance and patient safety to improve teamwork and enhance patient safety.

The low scores on communication factors (communication openness, feedback, and communication about error) may be suggest that there is still a strong culture of blame in Austria. It is well known that the cultural hierarchy in healthcare can lead to providers being afraid of speaking up or not being able to do their best [40]. These results can also partly explain health professionals’ perceptions of the communication factors, since Austrian hospitals are highly hierarchically organized. Communication is the main cause of most errors, as hospitals lack a systematic, universal method for accurately communicating important information [41]. Evidence-based tools and approaches have been developed to improve communication. For example, the implementation of SBAR (situation, background, assessment and recommendation), briefings and debriefings, Check back, Call out and Ask Me 3® are recommended to improve communication. Moreover, leaders can apply three key strategies to foster strong teams and good communication: 1. Making oneself approachable, 2. Inviting everyone into the conversation, and 3. Establishing shared goals [42]. Another factor leading to undesirable communication in healthcare is disrespectful behavior [40]. Therefore, it is essential to identify and address disrespectful behaviors in healthcare settings.

The low values for unit handoffs and transitions may be related to a lack of non-standard, non-written handoffs and transitions. Different documentation systems and technical terms may also be a reason for this result. To improve unit handoffs and transitions, standardized handoff protocols (e.g., I pass the baton [43]) should be used to overcome the loss of information between healthcare professionals. These protocols can promote more successful sharing of information and thus improve the outcomes for patients, providers, and organizations [43].

Both factors, hospital management support for patient safety and hospital handoffs and teamwork across hospital units, yielded a barely acceptable value at the hospital level. A meta-analysis showed that teamwork across hospital units had the best value in other studies [44]. However, the factor of hospital management support for patient safety has been identified as a weakness in many other studies [33, 45–48]. The low value of hospital management support for patient safety could perhaps be attributed to inappropriate leadership styles. As hospital management support has been identified as an essential factor for establishing and embedding a safety culture [27, 49, 50], we recommend a participative leadership style, which positively impacts PSC, for all Austrian hospitals [51–53].

Teamwork across hospital units is important because patients with typical chronic diseases see an average of 14 different doctors and require an average of 50 prescriptions per year [54]. To improve teamwork across hospital units, structured patient handoff programs, such as the I pass the baton program, should be introduced. It has been proven that this approach leads to significantly better patient outcomes [55]. In addition, new and innovative concepts of integrated care, such as case management or disease management, could be applied to improve teamwork across hospital units.

The outcome factor, frequency of event reporting, also showed a very low mean value. This result is very similar to that of other studies [33, 44, 56] and can be explained by the variable number of events reported, for which 68% of the staff stated that they had not reported a single event in the year before. This factor was moderately correlated with the patient safety grade, which means that it is vital that errors are reported to improve overall patient safety. However, another study suggests that reporting rates should not be used to assess hospital safety [57]. Different medical professionals focus on different types of safety incidents and, therefore, only report safety incidents that are important to them [57].

Increasing the values on all ten dimensions should be a top priority for hospitals in Austria. Failure to meet standards on these dimensions may have a huge impact on patients, staff, and hospitals.

The correlation analysis showed a positive correlation between the outcome variable patient safety grade and all PSC factors. This result indicates that the better the support of management, teamwork, communication, and feedback within the clinics, the better the patient safety in the hospital. If there is a culture of safety rather than a blame culture and if the hospital starts to deal with errors without sanctions, then the level of patient safety will improve. However, we found no correlation between the other two outcome variables (influence on patient safety and number of events reported) and PSC factors. Thus, the possibility of influencing patient safety may have no significant impact on PSC factors. Moreover, the number of reports had no influence on PSC factors. Other studies have shown that staff also enter trivial events into error reporting systems [58, 59], and only a few serious cases have been reported. Furthermore, error reporting systems have been proven to miss 90% of AEs that other methods could have detected, such as the Global Trigger Tool [11]. However, the number of reports entered in error reporting systems does not reflect the PSC in a hospital. The positive correlation between patient safety grade and frequency of event reporting suggests that reporting errors indirectly and positively affect patient safety.

Ensuring patient safety involves teamwork and only when everyone is committed to patient safety, can we achieve safe processes and good patient outcome performance [60–62]. It is evident that different professional groups have significantly different perceptions of PSC [63]. It is therefore essential to know which professional groups are more and which ones are less committed to factors of PSC. Analogous to these results, targeted interventions can then be implemented to improve PSC factors. We found significant differences between professional groups in seven out of ten PSC factors. Other studies have reported different perceptions of physicians and nurses regarding PSC [44, 47, 63]. This difference may be explained by the fact that nurses and doctors in the same team have different management structures [63]. These differences in perceptions of PSC among professional groups should be acknowledged to adequately generate interventions focused on improving patient safety.

The study provides the Austrian Federal Ministry for Social Affairs, Health, Care, and Consumer Protection with a solid basis for initiating and developing further measures to improve patient safety. Each hospital survey received its own results and individualized recommendations for action. The hospitals can now implement corrective measures based on individual results. Before implementing an intervention, the hospital should check whether there is an evidence-based implementation strategy for this intervention. After implementing the corrective actions, we recommend repeating patient safety measurements using the same instrument. This will allow hospitals to evaluate the effectiveness and efficiency of corrective actions. All hospitals outside this study can use CIs to estimate their PSC status.

Future research should interview health professionals to gain further insight into the underlying causes and possible explanations for this study’s findings. Interventions should be implemented to overcome these barriers. Moreover, this study should be repeated to verify the effectiveness of the interventions. Such a design would also provide a longitudinal view of the data.

Our study has several limitations. First, the results are comparable to international studies with unchanged factors only. Second, the response rate was not very high. However, we believe that the response rate had almost no impact on the results, as there was a large pool of health professionals (doctors, nurses, etc.). However, we have confirmed that our sample is well-suited for an overview study with archival analysis. Third, the present study only represents a snapshot of the PSC status in Austria and should be carried out repeatedly to consider the change over time.

## Conclusions

The PSC in Austria is low and needs improvement. We illustrate that all ten PSC factors have considerable potential to be improved from a hospital, unit, and outcome perspective. These results highlight the urgency of change in Austrian hospitals. However, evidence-based recommendations were suggested to improve the PSC in Austria in a targeted manner.

## Supporting information

S1 Dataset (XLSX)
